# Ungulate preference for burned patches reveals strength of fire–grazing interaction

**DOI:** 10.1002/ece3.12

**Published:** 2011-10

**Authors:** Brady W Allred, Samuel D Fuhlendorf, David M Engle, R Dwayne Elmore

**Affiliations:** Department of Natural Resource Ecology & Management, Oklahoma State UniversityStillwater, Oklahoma

**Keywords:** Behavior, conservation, disturbance, grassland, heterogeneity, pyric herbivory, tallgrass prairie

## Abstract

The interactions between fire and grazing are widespread throughout fire-dependent landscapes. The utilization of burned areas by grazing animals establishes the fire–grazing interaction, but the preference for recently burned areas relative to other influences (water, topography, etc.) is unknown. In this study, we determine the strength of the fire–grazing interaction by quantifying the influence of fire on ungulate site selection. We compare the preference for recently burned patches relative to the influence of other environmental factors that contribute to site selection; compare that preference between native and introduced ungulates; test relationships between area burned and herbivore preference; and determine forage quality and quantity as mechanisms of site selection. We used two large ungulate species at two grassland locations within the southern Great Plains, USA. At each location, spatially distinct patches were burned within larger areas through time, allowing animals to select among burned and unburned areas. Using fine scale ungulate location data, we estimated resource selection functions to examine environmental factors in site selection. Ungulates preferred recently burned areas and avoided areas with greater time since fire, regardless of the size of landscape, herbivore species, or proportion of area burned. Forage quality was inversely related to time since fire, while forage quantity was positively related. We show that fire is an important component of large ungulate behavior with a strong influence on site selection that drives the fire–grazing interaction. This interaction is an ecosystem process that supersedes fire and grazing as separate factors, shaping grassland landscapes. Inclusion of the fire–grazing interaction into ecological studies and conservation practices of fire-prone systems will aid in better understanding and managing these systems.

## Introduction

Fire and grazing affect a large proportion of the earth's ecosystems (Milchunas and Lauenroth 1993; Bond et al. 2005), playing a critical role in both establishment and maintenance of grasslands and savannas (Milchunas et al. 1988; van Langevelde et al. 2003; Anderson 2006). While fire and grazing affect ecosystem processes independently, the interaction between them may be more ecologically important than their independent effects. This interaction has been proposed as a single disturbance, pyric herbivory, defined as grazing driven by fire (Fuhlendorf et al. 2009). The fire–grazing interaction is described by positive and negative feedbacks in a tightly coupled fire–grazing system, creating new states and effects not present when the two processes are examined independently (Fuhlendorf and Engle 2004; Archibald et al. 2005). When fire occurs in patches across a landscape, herbivores preferentially select recently burned areas over areas with greater time since fire (Vinton et al. 1993; Sensenig et al. 2010). Due to the dependence of fuel accumulation on grazing pressure, probability of fire and fire behavior responds correspondingly to variation in herbivory (Leonard et al. 2010). These positive and negative feedbacks result in a complex disturbance interaction that is best expressed as spatiotemporal patterns across the landscape.

The fire–grazing interaction is dynamic in space and time, creating a shifting mosaic (Fuhlendorf and Engle 2004). This interaction shapes the landscape, creating heterogeneity at multiple scales (Fuhlendorf and Engle 2001; Archibald et al. 2005). Due to the complex spatiotemporal pattern, fire–grazing interactions are critical to grassland ecosystem structure and function. Variable vegetation structure associated with the fire–grazing interaction is important to biodiversity (Fuhlendorf et al. 2006), fire behavior (Kerby et al. 2007; Kirkpatrick et al. 2011), invasive species populations (Cummings et al. 2007), animal populations and communities (Fuhlendorf et al. 2010; Parrini and Owen–Smith 2010), and ecosystem processes (Anderson et al. 2006).

Referred to as the “magnet effect” by Archibald et al. (2005), burned areas attract grazing animals, resulting in heavy selection and use. This attraction to recently burned areas has been documented with numerous animal species throughout the globe (Pearson et al. 1995; Moe and Wegge 1997; Kramer et al. 2003; Klop et al. 2007; Murphy and Bowman 2007; Onodi et al. 2008). Although it is widely known that herbivores are attracted to burned areas, most large herbivore behavior studies do not include direct effects of fire, but focus instead on other abiotic (e.g., topography, temperature, climate, etc.) or biotic (e.g., forage quantity, predation, etc.) characteristics (e.g., Bailey et al. 1996; Fortin et al. 2003; de Knegt et al. 2007; Winnie et al. 2008; Beest et al. 2010). The influence of fire on site selection, in relation to other factors, is a key component of the fire–grazing interaction that is not well understood. While herbivore attraction to burned areas has been recognized, there is little work focused on the magnitude of the attraction as the context or mechanism of the fire–grazing interaction (but see Sensenig et al. 2010).

Our principal goal was to determine the strength of the fire–grazing interaction by examining the influence of fire on ungulate site selection across locations that varied in area and complexity, ranging from a large landscape with random fires to smaller landscapes with fixed fire patterns. To be clear, we do not directly assess the interaction itself (i.e., comparing systems with and without the interaction) but rather focus on understanding primary mechanisms of the fire–grazing interaction. The overall strength or significance of the fire–grazing interaction can be determined by examining how fire influences grazing behavior (the key link between fire and grazing). A pronounced and persistent influence will reveal a strong interaction, while a subtle or slight influence will indicate a weak interaction. Our specific objectives were to (1) compare ungulate preference for recently burned patches relative to the influence of other environmental factors, (2) compare that preference between native and introduced ungulate species, (3) test relationships between proportion of area burned and herbivore preference, and (4) determine forage quality and quantity as causal mechanisms of site selection. We show that fire is a primary driver in large herbivore behavior and that the fire–grazing interaction is an integral process within tallgrass prairies.

## Methods

This study was conducted at two locations within the Southern Great Plains, USA: The Nature Conservancy Tallgrass Prairie Preserve, north of Pawhuska, OK, USA and the Oklahoma State University Research Range, southwest of Stillwater, OK, USA. The vegetation at both sites is classified as tallgrass prairie with small patches of crosstimbers forest. Dominant grasses include *Andropogon gerardii* Vitman, *Schizachyrium scoparium* (Michx.) Nash, *Panicum virgatum* L., and *Sorghastrum nutans* (L.) Nash. Crosstimbers vegetation is dominated by *Quercus stellata* Wang. and *Q. marilandica* Münchh. Fire–grazing interactions are a dominant feature at both sites with spatially distinct patches burned within larger areas during both dormant and growing seasons (Fuhlendorf and Engle 2004; Hamilton 2007).

### Experimental design

The Tallgrass Prairie Preserve contains one large unit (9532 ha) that is grazed by native bison (*Bison bison*) and five smaller units (430–980 ha) grazed by introduced cattle (*Bos taurus*). Bison and cattle have access to all areas within their respective units (i.e., there are no interior fences). Bison are maintained in their unit throughout the year; herd size is approximately 2300 animals. Sex ratio of the bison herd is approximately seven females per male; ages of females range from 0 to 10 years, while males are 0–6 years. Herding and group sizes vary throughout the year; large, combined (bulls, cows, calves) groups are most common in summer months, while smaller, separated groups are present the rest of the year (Schuler et al. 2006). It is rare that female bison are found alone or grazing independently (B. Allred, personal observation). Cattle units are stocked with stocker steers approximately 1 year of age (mixed European breeds); cattle are present April through September. Cattle numbers vary with unit, ranging from 169 to 463 steers. Cattle often congregate in herds, similar but smaller than that of bison (B. Allred, personal observation). Bison and cattle are minimally handled and provided with no supplemental feed. All units are stocked with similar moderate stocking rates (bison: 2.1 AUM/ha; cattle: 2.4 AUM/ha).

**Figure 1:**
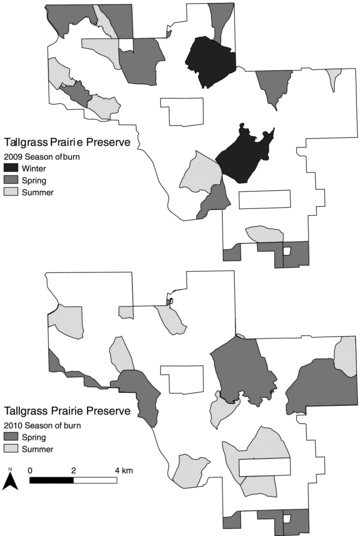
Illustration of patchy fire within the bison unit (9532 ha) at the Tallgrass Prairie Preserve, OK, USA. Map displays fires applied in 2009 and 2010. Spatially distinct patches are burned within the bison unit in spring, summer, and winter. Burn locations are not fixed and vary by year and season. Only perimeter fences are present, allowing bison free access to all burns. The fire–grazing interaction occurs as bison select between recently burned and areas with greater time since fire.

Approximately, one-third of the bison unit is burned annually. Burn patches vary in area (100–700 ha) and are located randomly across the landscape (noncontiguous, no fixed burn units; Fig. 1). About 80% of area burned occurs during the dormant season (40% in winter, 40% in late spring) and 20% during the growing season (Hamilton 2007). The variability in time since fire of patches ranges from 0 to 6 years. We manipulated the proportion of area burned within cattle units to examine the influence of relative burned area available on ungulate site selection. We assigned each cattle unit a fire patch size of 50 (i.e., half the unit is burned), 33, 25, 17, or 12% (see Fig. S1). In contrast to randomly located burned patches within the bison unit, location of patches in cattle units is fixed and contiguous. Variability in time since fire of patches ranges from 0 to 4 years and is dependent upon proportion of area burned.

We fitted bison and cattle with global positioning systems (GPS; GPS7000MU & GPS3300L, Lotek Wireless, Newmarket, Canada). We deployed GPS collars on seven bison from November 2008 through November 2010 (batteries replaced and new animals chosen in November 2009) and five cattle (one per unit) from April through September of 2009 and 2010 (batteries replaced and new animals chosen in April 2010). We recorded location information of each animal at frequencies ranging from 12 min to 1 h.

To further understand the influence of fire on ungulate site selection at finer spatial scales, we used two units (65 ha each) grazed by cattle at the Oklahoma State University Research Range. As with the Tallgrass Prairie Preserve, only unit perimeter fences are present and animals are free to roam within their respective units. Units are equally stocked (3.0 AUM/ha) with cattle (European breeds, yearlong cow–calf operation). One-sixth of each unit is burned in the late dormant season and an additional one-sixth during the growing season (Fig. S1). Variability in time since fire ranges from 0 to 3 years. We fitted cattle with GPS collars (GPS3300LR, Lotek Wireless); we deployed GPS collars on individual cattle (one per unit) from August 2007 through December 2009. We recorded location information at a frequency of 5 min. Collars were retrieved every 6 weeks to replace batteries. We omitted data from days in which animal behavior was influenced by human activity, for example, general animal husbandry practices. Though smaller in size and animal numbers than other sites, cattle were often found congregated and grazing together (B. Allred, personal observation).

### Spatial data

Animal location data were differentially corrected with stationary GPS data obtained from their respective location; corrected data were imported into a spatially enabled database (PostgreSQL/PostGIS). We mapped unit perimeter, fire history, water sources, and woody vegetation at all sites with handheld GPS units, aerial and satellite imagery, and U.S. Geological Survey 7.5 min topographic maps. We obtained topography information (elevation, slope, aspect) from digital elevation models for each location. Aspect data were transformed with simple trigonometric functions by creating two variables, northing = cosine(aspect) and easting = sin(aspect). Variability of time since fire, elevation, water sources, and woody vegetation of the bison unit at the Tallgrass Prairie Preserve is shown in Figures S2–5. Variability of cattle units at the Tallgrass Prairie Preserve is similar to the bison unit; variability of cattle units at the Oklahoma State University Research Range is reduced due to smaller size.

### Objective one

To compare the influence of time since fire relative to other environmental factors, we estimated resource selection functions (Boyce et al. 2002) for animals at each location. We established three random points for each observed location to provide estimates of available conditions across the landscape. We first tested whether animals used recently burned areas more than random; we compared the number of randomly placed points to recorded locations in areas that were 6 months since fire using a *t*-test. Distance to water, distance to fire patch edge, fire patch area, elevation, slope, northing, easting, and time since fire were associated with animal locations and established random points. We created resource selection functions using combinations of environmental factors for each site. Model parameter selection was based on knowledge of bison and cattle behavior and availability of data, either collected or remotely sensed. Crude protein and biomass data (discussed below) were not included in resource selection functions as they were sampled at only one site, within a narrower time frame and at a broader sampling frequency than animal location data. Although reviewers raised this concern, we show that using time since fire is satisfactory, as it is correlated with both crude protein and aboveground biomass. Because we were specifically interested in the influence of time since fire of burn patches, we included interaction terms for time since fire with all other variables (i.e., time since fire × distance to water, time since fire × slope, etc.). In all models with interaction terms, we included main effects of both variables. To compare influence of environmental factors, and to more easily interpret interaction terms, we standardized variables by subtracting their mean and dividing by their standard deviation (Gelman and Hill 2007). To account for correlation within an individual animal and among animals, individuals were included as a random intercept in logistic regressions; for cattle at the Tallgrass Prairie Preserve, individuals were also nested within their respective unit (Gillies et al. 2006). We compared and ranked various resource selection functions using Akaike information criterion (AIC; Burnham and Anderson 2002). We used bootstrapping procedures to estimate precision of resource selection coefficients and to test differences in influence of environmental factors within species at each research location. We compared coefficients after calculating confidence intervals (95%) from 1000 iterations of randomly sampled datasets; coefficients were considered different if confidence intervals did not overlap.

### Objective two

We used the bison and cattle units at the Tallgrass Prairie Preserve to compare preference for recently burned areas (as well other environmental factors) between native (bison) and introduced (cattle) ungulates in tallgrass prairie. To appropriately compare selection between the two, we reduced bison location data to match that of cattle (April–September, as well as frequency of GPS fix). We estimated separate resource selection functions for each species using top-ranked models from objective one. We used bootstrapping procedures to estimate precision of resource selection coefficients and to test differences between species. We compared coefficients between species after calculating confidence intervals (95%) from 1,000 iterations of randomly sampled datasets; coefficients were considered different if confidence intervals did not overlap.

### Objective three

We examined the influence of proportion of area burned on preference for recently burned patches using cattle units at the Tallgrass Prairie Preserve (varying from 50 to 12% burned). We estimated separate resource selection functions for each fire patch size, following procedures in objective one. We used linear regression to determine a relationship between proportion burned and herbivore preference for recently burned areas.

### Objective four

We examined the response of forage quality and quantity to the fire–grazing interaction within cattle units of the Oklahoma State University Research Range. We harvested aboveground plant tissue (live and dead combined) from four randomly placed 0.10 m^2^ plots in patches that varied in time since fire. We collected samples every 2 weeks from April through November 2009. After drying samples to a constant mass, we recorded the weight of each sample and determined percent crude protein using a dry combustion analyzer (LECO Corp., St. Joseph, MI). We used linear regression to test relationships of crude protein and aboveground biomass to time since fire. We performed all analyses using R (R Development Core Team 2010) with additional use of the *lme4* package for mixed effects resource selection functions (Bates and Maechler 2010), and *doMPI* (Weston 2009), *foreach* (Revolution Computing 2009), and *Rmpi* (Yu 2010) packages for high-performance computing.

## Results

**Figure 2:**
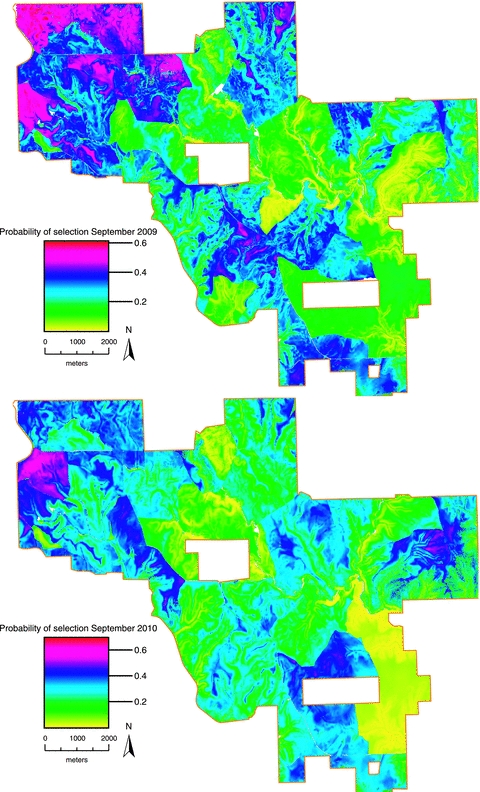
Relative probability of site selection by bison at the Tallgrass Prairie Preserve, OK, USA, for September 2009 and 2010. Probabilities presented as a function of parameters in Table 1. Solid orange lines represent perimeter fences. See Figure 1 for recently burned areas. Bison prefer recently burned areas; probabilities change as fire moves around the landscape.

Animals at each research location used recently burned areas more than random (*P* < 0.05). Common environmental factors that influence ungulate site selection were of lesser influence than time since fire (objective one; Table 1). Of resource selection functions examined for bison, the model that contained interaction terms of time since fire with all variables less northing and easting, had the best fit based on AICs; (Table S1). Based on resource selection coefficients, primary drivers of bison site selection were time since fire (selecting recently burned areas) and avoiding woody vegetation (Table 1). Bison also avoided steeper slopes and larger fire patches. Bison selected areas closer to water and fire patch edge, but both had a small influence relative to other variables. Interactions of time since fire with other variables show that fire is critical to understanding most aspects of grazing behavior. The influence of time since fire increased as slope, distance to fire patch edge, fire patch area, and elevation increased. Conversely, the influence of time since fire decreased as distance to water increased and as woody vegetation became present. The probability of selection for bison at the Tallgrass Prairie Preserve, based upon parameters in Table 1, is displayed in Figure 2.

**Table 1:** Estimated resource selection function coefficients for bison and cattle at the Tallgrass Prairie Preserve, OK, USA and cattle at the Oklahoma State University Research Range, OK, USA. Model parameters include distance to water (m), distance to fire patch edge (m), slope (%), elevation (m), fire patch area (ha), northing and easting (°; both derivatives of aspect), woody vegetation, and time since fire (days). Standardized variables shown for coefficient comparison. Letters indicate overlap in confidence interval (95%) within species and research location; confidence intervals calculated using bootstrapping procedures (1000 iterations)

Bison, Tallgrass Prairie Preserve	Estimate	SE	*Z*-value	*P*
Intercept	−1.2901	0.0058	−220.34	<0.01
Time since fire	−0.7373	0.0033	−222.68	<0.01
Distance to water	−0.0100^a^	0.0023	−4.62	<0.01
Slope	−0.4370	0.0033	−130.67	<0.01
Distance to patch edge	−0.0133^a^	0.0027	−4.9	<0.01
Woody vegetation	−1.0759	0.0178	−60.33	<0.01
Elevation	0.1604	0.0025	62.42	<0.01
Patch area	−0.3460	0.0034	−100.85	<0.01
Time since fire × distance to water	0.0952	0.0024	38.83	<0.01
Time since fire × slope	−0.1523	0.0039	−38.15	<0.01
Time since fire × distance to patch edge	−0.1161	0.0031	−37.36	<0.01
Time since fire × woody	0.0521	0.0217	2.40	0.01
Time since fire × elevation	−0.1356	0.0027	−49.09	<0.01
Time since fire × patch area	−0.5156	0.0054	−95.27	<0.01

Cattle, Tallgrass Prairie Preserve	Estimate	SE	*Z*-value	*P*
Intercept	3.4719	0.4446	7.81	<0.01
Time since fire	−0.6959	0.0041	−168.44	<0.01
Distance to water	−0.0214	0.0032	−6.68	<0.01
Slope	−0.2079	0.0034	−60.31	<0.01
Distance to patch edge	−0.0798	0.0030	−26.61	<0.01
Woody vegetation	0.9805	0.0190	51.53	<0.01
Elevation	0.0121	0.0037	3.27	<0.01
Northing	−0.0075^a^	0.0025	−2.97	<0.01
Easting	−0.0077^a^	0.0025	−3.04	<0.01
Time since fire × distance to water	−0.1661	0.0041	−39.94	<0.01
Time since fire × slope	−0.1800	0.0045	−39.28	<0.01
Time since fire × distance to patch edge	0.0317	0.0029	10.79	<0.01
Time since fire × woody	0.3297	0.0182	18.07	<0.01
Time since fire × elevation	−0.0558	0.0045	−12.25	<0.01

Cattle, Research Range	Estimate	SE	*Z*-value	*P*
Intercept	−1.3277	0.0032	−413.47	<0.01
Time since fire	−0.7614	0.0033	−224.54	<0.01
Distance to water	0.1398	0.0028	48.69	<0.01
Slope	−0.1010	0.0030	−33.39	<0.01
Woody vegetation	0.5993	0.0081	−73.24	<0.01
Northing	0.0151^a^	0.0026	5.62	<0.01
Easting	0.0061^a^	0.0026	2.28	0.02
Time since fire × distance to water	−0.0387^b^	0.0029	−13.05	<0.01
Time since fire × slope	−0.0292^b^	0.0033	−8.78	<0.01
Time since fire × woody	0.2355	0.0088	26.72	<0.01

Time since fire also was a primary driver in site selection by cattle at the Tallgrass Prairie Preserve (Table 1). The combination of interaction terms of time since fire with most other variables (less northing and easting) had the best fit based on AICs (Table S2). Cattle selected recently burned patches, minimizing the amount of time since fire. In contrast to selection behavior of bison, however, cattle preferred woody vegetation over all other attractants. Cattle selected areas closer to water and patch edge, and avoided steeper slopes. Interactions of time since fire with other predictors again indicate the complexity of the influence of fire on site selection. At the Oklahoma State University Research Range, where unit size is smaller than other research locations, the preference for recently burned areas was also strong (Table 1). Of models examined, the combination of interaction terms of time since fire with most variables (less northing and easting) had the best fit based on AICs similar to cattle in larger units (Table S3). Similar to other sites, cattle preferred recently burned areas. Cattle were also attracted to woody vegetation. As with other research locations described, the interactions of time since fire with other factors were present. Preference for recently burned areas was a primary driving force in site selection, with greater influence than other factors (objective one).

Comparison of bison and cattle selection revealed similar and contrasting preferences (Table 2). After appropriately matching data, most coefficients were similar in preference or avoidance (indicated by sign of coefficient, ±) to population resource selection functions (created using full datasets, Table 1) but varied in magnitude. Selection changed for distance to water in bison (minimized distance to maximized distance) and cattle (minimized distance to maximize distance), and elevation (preferred higher elevations to avoided higher elevations) in cattle. While both species had strong preferences for recently burned areas, the magnitude of preference in native bison was greater than introduced cattle (objective two).

**Table 2:** Estimated resource selection function coefficients comparing native bison and introduced cattle at the Tallgrass Prairie Preserve, OK, USA. Data were reduced to the months of April–September and equal frequency sampling to appropriately compare selection between the two species. Model parameters include distance to water (m), distance to fire patch edge (m), slope (%), elevation (m), fire patch area (ha), northing and easting (°; both derivatives of aspect), woody vegetation, and time since fire (days). Standardized variables are shown for coefficient comparison. Letters indicate overlap in confidence interval (95%) between bison and cattle; confidence intervals calculated using bootstrapping procedures (1000 iterations)

	Bison	Cattle
Intercept	−1.8795	3.2734
Time since fire	−1.6072	−0.7438
Distance to water	0.0724	0.0075
Slope	−0.5338	−0.2242
Distance to patch edge	−0.0425	−0.0990
Woody vegetation	−0.8216	1.1566
Elevation	0.2095	−0.0531
Patch area	−0.4735	−
Northness	−	−0.0170
Eastness	−	−0.0040
Time since fire × distance to water	0.1656^a^	0.1534^a^
Time since fire × slope	−0.2554	−0.2097
Time since fire × distance to patch edge	−0.2004	0.0453
Time since fire × woody	0.3705^b^	0.3690^b^
Time since fire × elevation	−0.0446	−0.1096
Time since fire × patch area	−0.7287	−

Resource selection functions for individual cattle units that varied in proportion and size of fire patch also displayed a strong influence of fire on site selection. Best-fit models for cattle units varied by individual units, but consistently included interactions of time since fire with other variables (Table S4). Similar to the overall population model (in which cattle units were analyzed collectively), cattle primarily selected for recently burned and woody vegetation areas (Table 3). The proportion of area burned did not correlate with herbivore preference for burned areas. Coefficients for time since fire varied among cattle units, but there was no relationship with proportion burned (*P* > 0.05; objective three), that is, preference for burned areas was not significantly altered if half or one-eighth of the area was burned.

**Table 3:** Estimated resource selection function coefficients for cattle units that varied in proportion of area burned at the Tallgrass Prairie Preserve, OK, USA. Model parameters include distance to water (wtr; m), distance to fire patch edge (m), slope (slp; %), elevation (elev; m), northing (north) and easting (east; °, both derivatives of aspect), woody vegetation (wdy), and time since fire (tsf; days). Standardized variables are shown for coefficient comparison

Proportion burned	Time since fire	Water	Slope	Edge	Woody	Elevation	Northness	Eastness
50	−0.8152	−0.1928	−0.2224	−0.1824	−0.2644	0.4938	−	−
33	−0.9401	0.1866	−0.0837	−0.2114	2.9839	0.0392	0.0182	−0.0171
25	−0.7408	0.0663	−0.1999	−0.1733	1.1045	−0.2263	−	−
17	−0.8191	−0.0493	−0.0155	−0.4602	2.5479	0.1785	−0.0466	−0.0192
12	−0.5010	−0.2257	−0.2363	−0.1436	1.1764	0.1511	−	−
Size	tsf × wtr	tsf × slp	tsf × edge	tsf × wdy	tsf × elev	tsf × north	tsf × east	
50	−	−0.0874	−0.3173	−0.1095	0.1265	−	−	
33	0.0391	0.0062	−0.3838	0.6220	−0.0375	−	−	
25	−0.0716	−0.0267	−0.1980	0.2719	0.0282	−	−	
17	−0.0531	−0.0232	−0.5955	−0.1584	0.1120	−0.0338	−0.0115	
12	−0.1726	−0.0789	−0.1271	−0.3816	0.1483	−	−	

**Figure 3:**
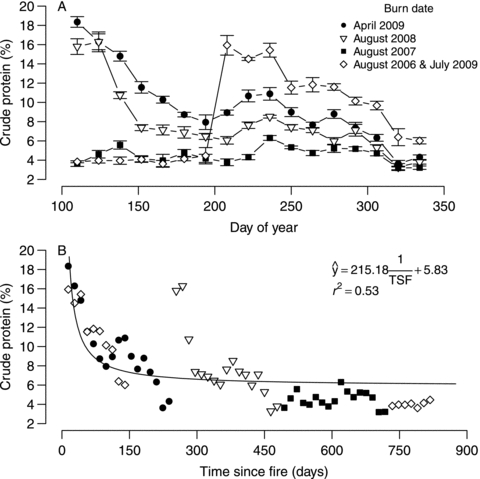
Crude protein (%) of tallgrass prairie vegetation from April to December 2009 at the Oklahoma State University Research Range, OK, USA. Symbols are means (*n*= 4) representing patches that vary in the amount of time since fire; error bars are one standard error. (A) Crude protein shown by day of year. (B) Crude protein as determined by the amount of time since fire (days).

**Figure 4:**
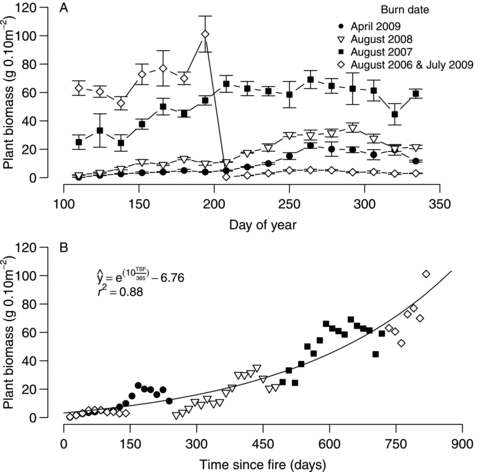
Aboveground plant biomass (g 0.10 m^−2^) of tallgrass prairie vegetation from April to December 2009 at the Oklahoma State University Research Range, OK, USA. Symbols are means (*n*= 4) representing patches that vary in the amount of time since fire; error bars are one standard error. (A) Aboveground plant biomass shown by day of year. (B) Aboveground plant biomass as a function of time since fire (days).

Forage quality and quantity of patches were dependent upon time since fire (objective four). Crude protein of patch vegetation was greatest in the most recently burned area regardless of season of burn (Fig. 3A and 3B). Forage quality decreased with time since fire (*P* < 0.05); at the end of sampling, forage quality within recently burned areas was nearly double that of other areas. In contrast to forage quality, forage quantity was lowest in recently burned areas and increased with time since fire (Fig. 4A and 4B; *P* < 0.05). A tradeoff between forage quality and quantity was present; areas with highest quality forage had the least quantities.

## Discussion

The ecological interactions between fire and grazing are important and have a defining role across complex landscapes (Archibald et al. 2005; Leonard et al. 2010; Sensenig et al. 2010). By specifically quantifying the influence of fire on ungulate site selection, we were able to measure the primary mechanism responsible for the fire–grazing interaction and better understand the role that fire and grazing play within these systems. The broad-scale observational and experimental work in this study reveals that fire has a strong influence on animal behavior and that the interaction between fire and grazing itself is strong. The amount of time since a particular area has burned becomes the critical link between fire and grazing, as it is a driving force in site selection. We found that the simple presence of fire is less significant than the pattern or heterogeneity resulting from patch fires, which forms the mosaic that influences animal selection. If fire occurs homogeneously across the complete area available to grazing animals, the interactions between fire and grazing cannot occur.

For herbivores in our study, time since fire ultimately changed how animals distributed themselves, a key component to the fire–grazing interaction. Time since fire had a greater influence than slope or distance to water, two factors that have been shown to primarily determine site selection of bison and cattle (Bailey et al. 1996). Woody vegetation, on the other hand, appeared to be the primary determining factor of site selection, even greater than fire. Native bison avoided areas with trees, while domestic cattle preferred them. These dissimilarities may be attributed to differences in thermal regulation between the two species (Christopherson et al. 1979), with woody canopy cover providing shade from solar radiation, particularly for cattle. It is often speculated that bison do not seek cover from solar radiation, as animals are adapted to temperature extremes of the Great Plains (Gogan et al. 2010). If true, there is likely little need for bison to select wooded areas, as vegetation is often different and reduced in quantity (Limb et al. 2010). Bison also preferred smaller burned patches over larger ones. As suggested by a reviewer, examining and incorporating other environmental variables deepens the definition and understanding of the fire–grazing interaction. It is not just the amount of time since fire that determines response but a suite of variables that influence one another. In particular, patch size contributes to grazing pressure (density of herbivores) of a recently burned patch, which can maintain vegetation characteristics to which grazers are attracted (high forage quality). Furthermore, by investigating the interaction of time since fire with other variables within resource selection functions, we show the complexity and connectedness of fire and grazing. For example, as time since fire increases, distance to patch edge becomes more important. Animals are more likely to stay closer to patch edges when in areas with greater time since fire, presumably to stay closer to preferred burned patches. Additionally, as slope increases, the magnitude of time since fire becomes greater. Animals will likely only select areas with steeper slopes if it has been recently burned. These interactions within selection decisions reinforce the ability of fire to modify behavior and the importance of studying the fire–grazing interaction.

The ability for fire to be a strong influence in herbivore behavior has many potential ecological consequences. The attraction to fire creates the fire–grazing interaction, which shapes the system, creates heterogeneity, influences ecosystem processes, and determines plant and animal populations and distributions (Archibald et al. 2005; Fuhlendorf et al. 2006; Leonard et al. 2010). In addition to site selection, fire may alter other individual behavior characteristics not studied in this paper, such as residence time, movement tortuousity, or traveling velocity (Kerby 2002), changing how animals interact with and gather information from the landscape. Understanding the interaction of fire and grazing may also demonstrate evolutionary mechanisms and history. Differences in the attraction to fire have been shown between foregut and hindgut fermenters, the former more attracted to fire and becoming more dominant during increased fires prior to the Pleistocene (Sensenig et al. 2010). With so many far-reaching effects, the fire–grazing interaction is to be considered an integral process of fire-prone systems.

The mechanisms of the fire–grazing interaction occur at multiple scales. At broad scales, fire and grazing must be present and able to influence one another (i.e., patchy fire; herbivores need to be able to select among burned and unburned areas). At finer scales, localized mechanisms attract animals to burned areas. Forage quality of plants in recently burned areas can be two to three times greater than areas with more time since fire (see also Sensenig et al. 2010). In tallgrass prairie, areas that were burned within a year had higher crude protein than areas with greater time since fire. As the growing season progressed, differences lessened and forage quality became more similar due to plant maturation. An additional fire in the middle of the growing season increased forage quality and was again greater than other available areas. These spikes in nutritional content, created by fire and subsequent grazing, can be vital for the productivity of grazing animals within the system (Verweij et al. 2006; Parrini and Owen–Smith 2010). With patch fires occurring regularly and throughout the landscape, high-quality forage is readily available and maintained. Patch size will then play an important role in the maintenance of burned areas. Due simply to size, smaller patches will have greater grazing pressure (greater density of herbivores) and will be easier for animals to keep in a short developing state of high nutritional value, similar to grazing lawns (Waite 1963). This is the likely reason bison preferred smaller patches over larger ones. This maintenance of the burn patch is also shown by the preservation of higher forage quality and low biomass well past the growing season (December). The spatial heterogeneity of forage quality created by patchy fire and subsequent grazing is also primary mechanism of the fire–grazing interaction. The continual preference for burned areas is due to increased nutritional content in post fire regrowth (Hobbs et al. 1991; van de Vijver et al. 1999).

Along with site selection and other behavior attributes, the fire–grazing interaction may modify foraging strategies. Though high-quality forage is readily available, grazing animals must also make decisions regarding the tradeoff between quality and quantity (Demment and van Soest 1985; Senft et al. 1987). In recently burned areas, where quantity is low, intake rates are constrained by plant cropping, whereas in areas with greater time since fire, intake rates become constrained by handling or processing (Spalinger and Hobbs 1992). Additionally, as plant biomass increases or matures, quality and digestibility decline (van Soest 1994). Such tradeoffs have been resolved by showing that grazing animals maximize energy intake by selecting for intermediate levels of vegetation quantity (Fryxell 1991; Mueller et al. 2008). Within the Serengeti, Wilmshurst et al. (1999) showed that wildebeest (*Connochaetes taurinus*) selected for intermediate biomass at broader landscapes scales, but not at finer local scales. In contrast, the findings presented here show that these grazing animals are primarily selecting recently burned patches, which contain the lowest amounts of biomass but highest amounts of protein. Decisions between forage quantity and quality will ultimately vary, depending upon the type of herbivore, resource availability, scale, etc. Due to metabolic requirements and animal physiology, larger herbivores may prefer both burned and unburned areas, while smaller animals may exclusively prefer burned areas (Wilsey 1996; van de Vijver et al. 1999; Sensenig et al. 2010).

The attraction of grazing animals to burned areas and the subsequent fire–grazing interaction are not phenomena restricted to North American grasslands, but are ecological processes that occur globally (Table S5). Magnitude of the attraction to burned areas and establishment of the fire–grazing interaction can be expected to differ across systems and species (see Klop et al. 2007; Bleich et al. 2008). The influence of environmental variables on herbivore behavior will depend upon their distribution and complexity across the landscape, for example, the influence of water is likely to be more influential in arid regions. Although predators are not present in the tallgrass prairie of this study, they would also play an important role in herbivore site selection. Herbivores may find refuge in recently burned areas, as visibility is increased and predators may be noticed more easily (Valeix et al. 2009; Eby 2010); but visibility of prey is also increased and may assist in predation. While the strength of the fire–grazing interaction may vary across systems, the interaction is likely to be present to some degree, influencing ecosystem structure and function.

Many fire-dependent systems, particularly grasslands and savannas, are endangered worldwide (Hoekstra et al. 2005). While conservation goals within these systems frequently involve restoring critical ecosystem processes, including fire and grazing (Hutto 2008; Sanderson et al. 2008), the importance of fire is often underrepresented (Bowman et al. 2009). Our findings contribute to the importance of fire within the ecosystem and support that fire and grazing are a coupled or single disturbance; their interaction may be just as vital for the conservation of fire-prone systems (Archibald et al. 2005; Fuhlendorf et al. 2009). Using knowledge from historical disturbance patterns, we can develop more effective land management and conservation strategies to preserve these endangered systems and their inherent processes. Furthermore, we show that the evolutionary disturbance patterns created by fire and grazing can be restored on working landscapes (domestic livestock production on small parcels). While there are differences between domestic and native or wild herbivores, using fire and grazing to manage livestock can help restore the defining role of these interactions, as well as critical processes that contribute to biodiversity and ecosystem function (Fuhlendorf and Engle 2001).

The fire–grazing interaction, however, is not simply a management tool for conservation, but an inherent ecological process of fire-prone systems. Simplifying or overlooking this interaction leads to an incomplete understanding of the effects of fire and herbivory (Fuhlendorf et al. 2009). Our data show that the time since an area has burned is a primary driver of ungulate behavior. Animals selectively prefer recently burned areas and avoid areas with greater time since fire. This preference establishes the fire–grazing interaction, creating new conditions and effects that are not present when investigating fire or grazing independently. Though the magnitude of this preference was not as influential as woody vegetation, it is high and greater than other environmental predictors, indicating a strong interaction between fire and grazing. Incorporating and accounting for the fire–grazing interaction in ecological studies and conservation will continue to improve our knowledge of these disturbances. Further study of the mechanisms of this interaction, as well as its influence on other ecosystem processes (e.g., nutrient flow, trophic interactions, primary productivity, etc.) is necessary to better understand fire-dependent landscapes.

## Supporting Information

**Figure S1.** Illustration of fire patches for cattle units at Tallgrass Prairie Preserve and Oklahoma State University Research Range.

**Figure S2.** Time since within the bison unit at the Tallgrass Prairie Preserve.

**Figure S3.** Elevation within the bison unit at the Tallgrass Prairie Preserve.

**Figure S4.** Distribution of water sourceswithin the bison unit at the Tallgrass Prairie Preserve.

**Figure S5.** Distribution of woody vegetation within the bison unit at the Tallgrass Prairie Preserve.

**Table S1.**.Models and AIC information for resource selection function of bison at Tallgrass Prairie Preserve.

**Table S2.**.Models and AIC information for resource selection function of cattle at Tallgrass Prairie Preserve.

**Table S3.**. Models and AIC information for resource selection function of cattle at Oklahoma StateUniversity Research Range.

**Table S4.**. Models and AIC information for resource selection function of cattle with respect to proportion burned at Tallgrass Prairie Preserve.

**Table S5.**. List of global studies examining the fire–grazing interaction.
