# Supplementary material for: Ungulate preference for burned patches reveals strength of fire–grazing interaction
Source: Ecol Evol. 2011 Oct;1(2):132–44. doi: 10.1002/ece3.12 (PMC3287302; doi:10.1002/ece3.12)
Supplement: Supplementary file 1 [file ece30001-0132-SD1.pdf]

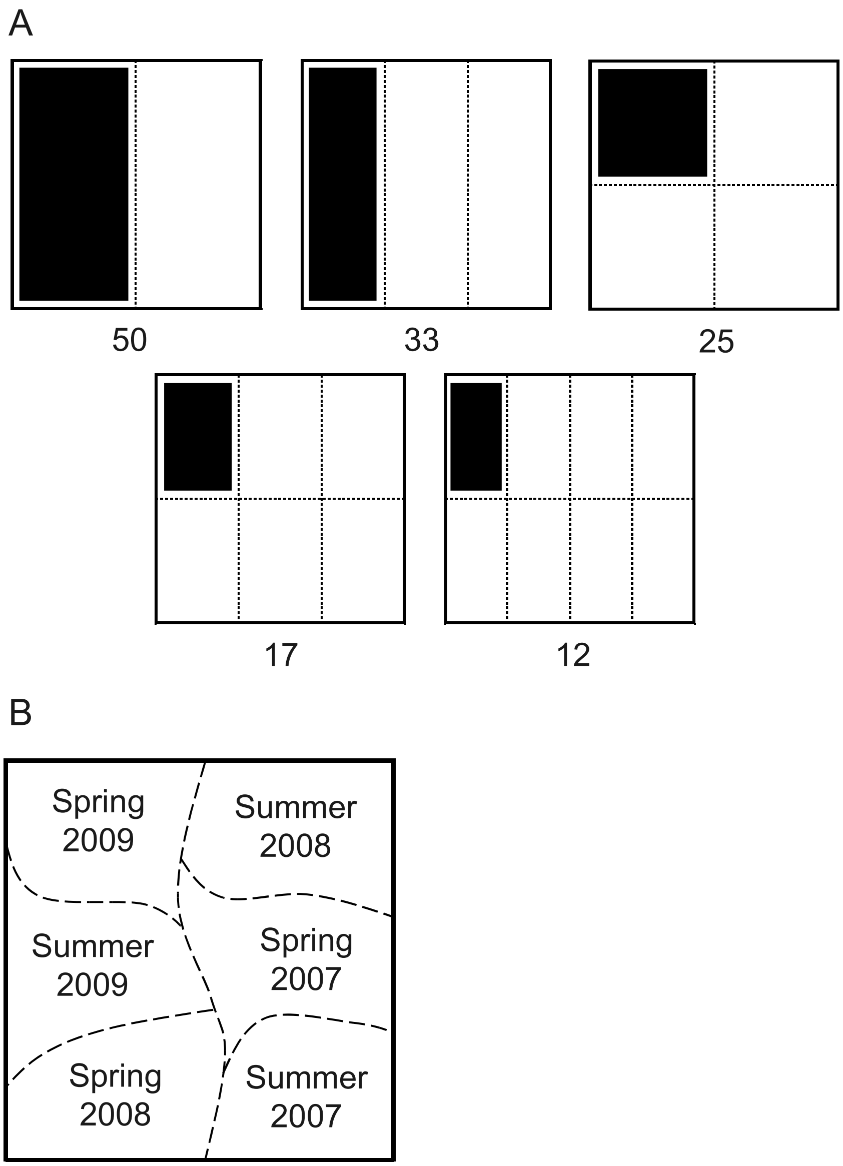


Figure S1: Illustration of cattle units at the Tallgrass Prairie Preserve, OK, USA and the Oklahoma State University Research Range, OK, USA. Only perimeter fences are present in units at both locations, allowing animals free access to burns. The fire-grazing interaction occurs as animals select between recently burned areas and areas with greater time since fire.

A) Units at the Tallgrass Prairie Preserve range in area from 430-980 ha; spatially distinct patches are burned within units. Size of five burn units was manipulated to include 50, 33, 25, 17, and 12\% of total area. Darkened areas in a unit represent a recently burned patch. Fire histories vary with each unit ranging from 0 to 4 years since fire.

B) Units at the Oklahoma State University Research Range are 65 ha; spatially distinct patches are burned within units. Text indicates burn sequence from summer 2007 through summer 2009.

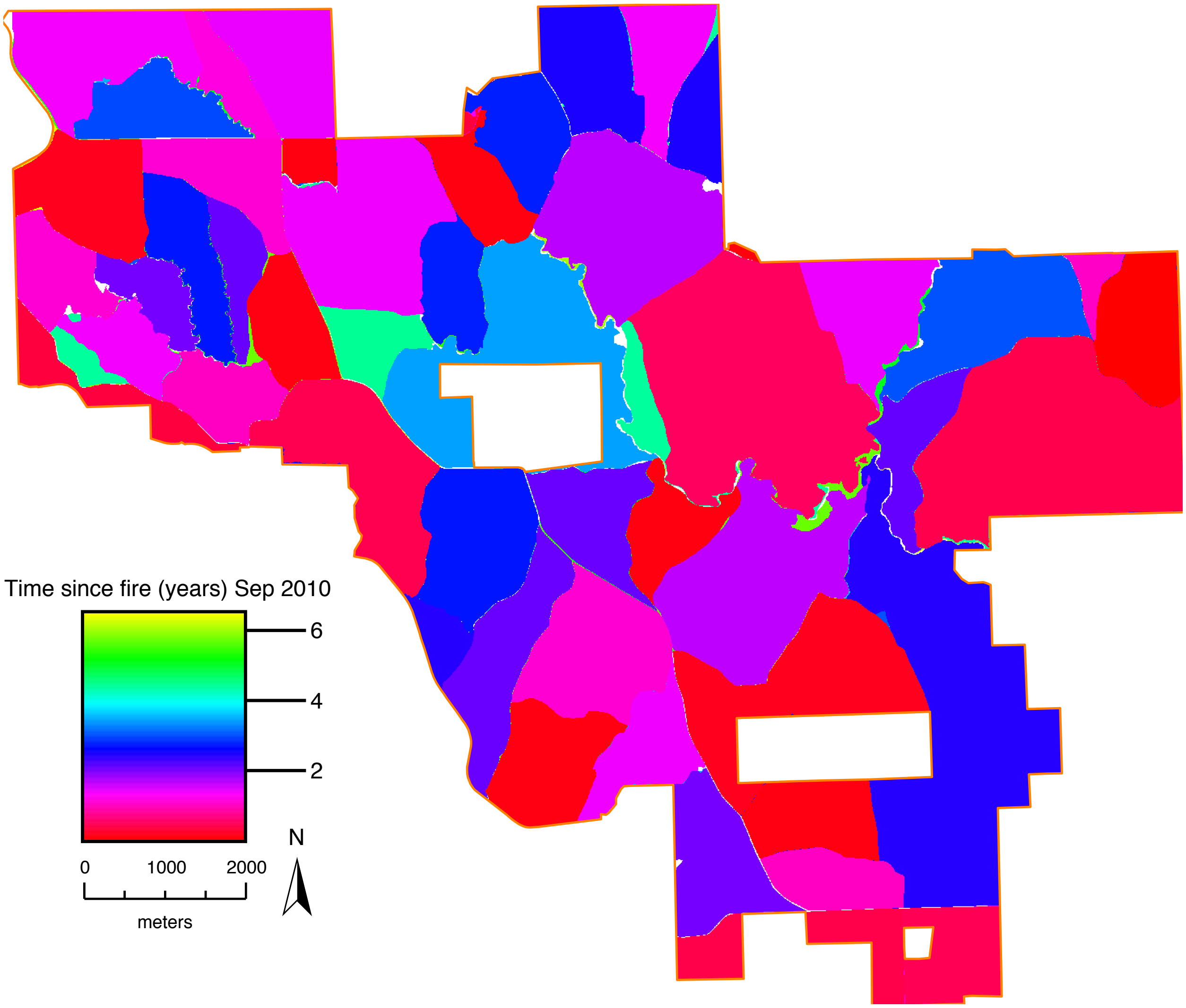


Figure S2: Range of time since fire (years) within the bison unit (9532 ha) at the Tallgrass Prairie Preserve, OK, USA. Solid orange lines represent perimeter fences.

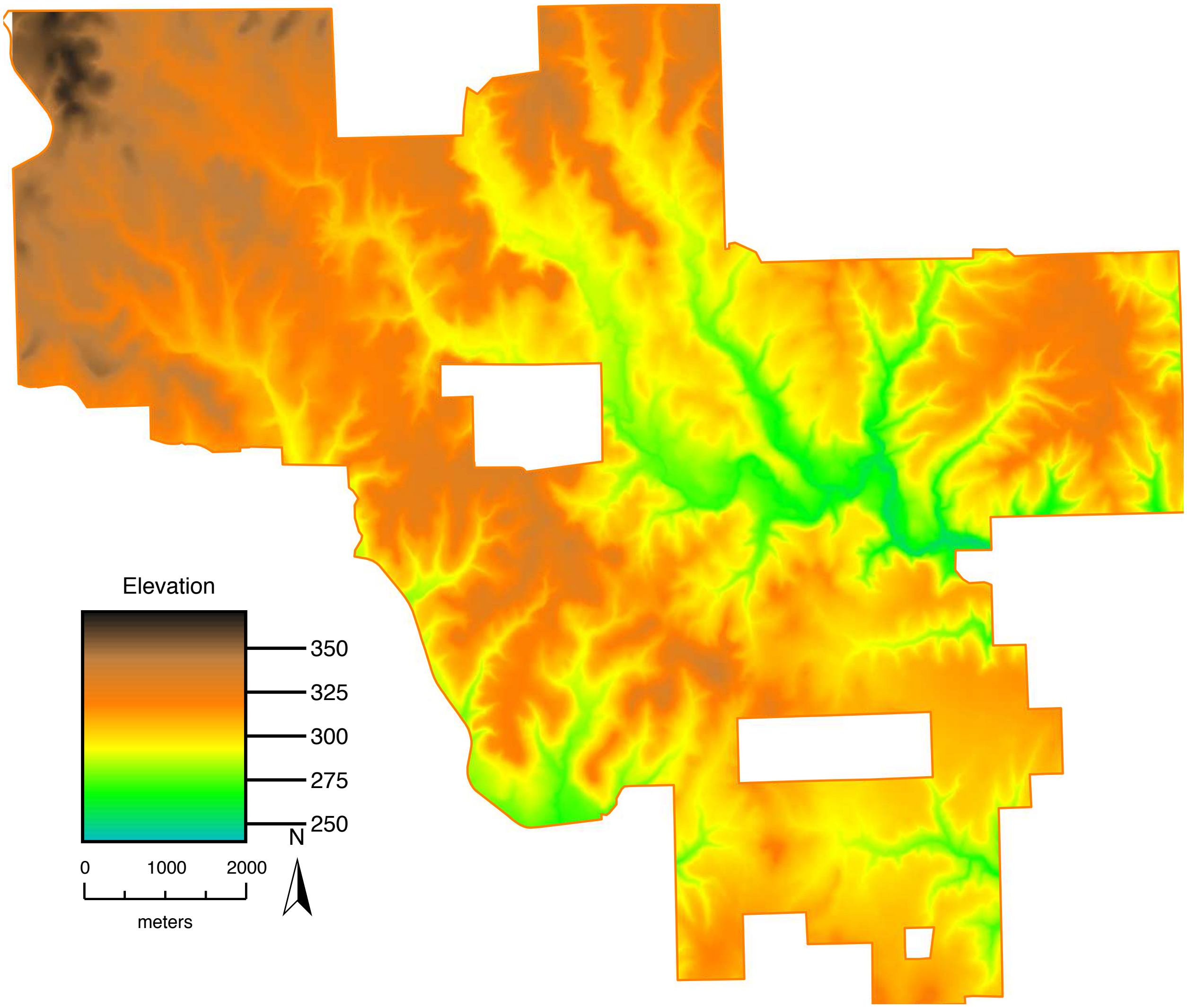


Figure S3: Elevation (meters) of the bison unit (9532 ha) at the Tallgrass Prairie Preserve, OK, USA. Solid orange lines represent perimeter fences.

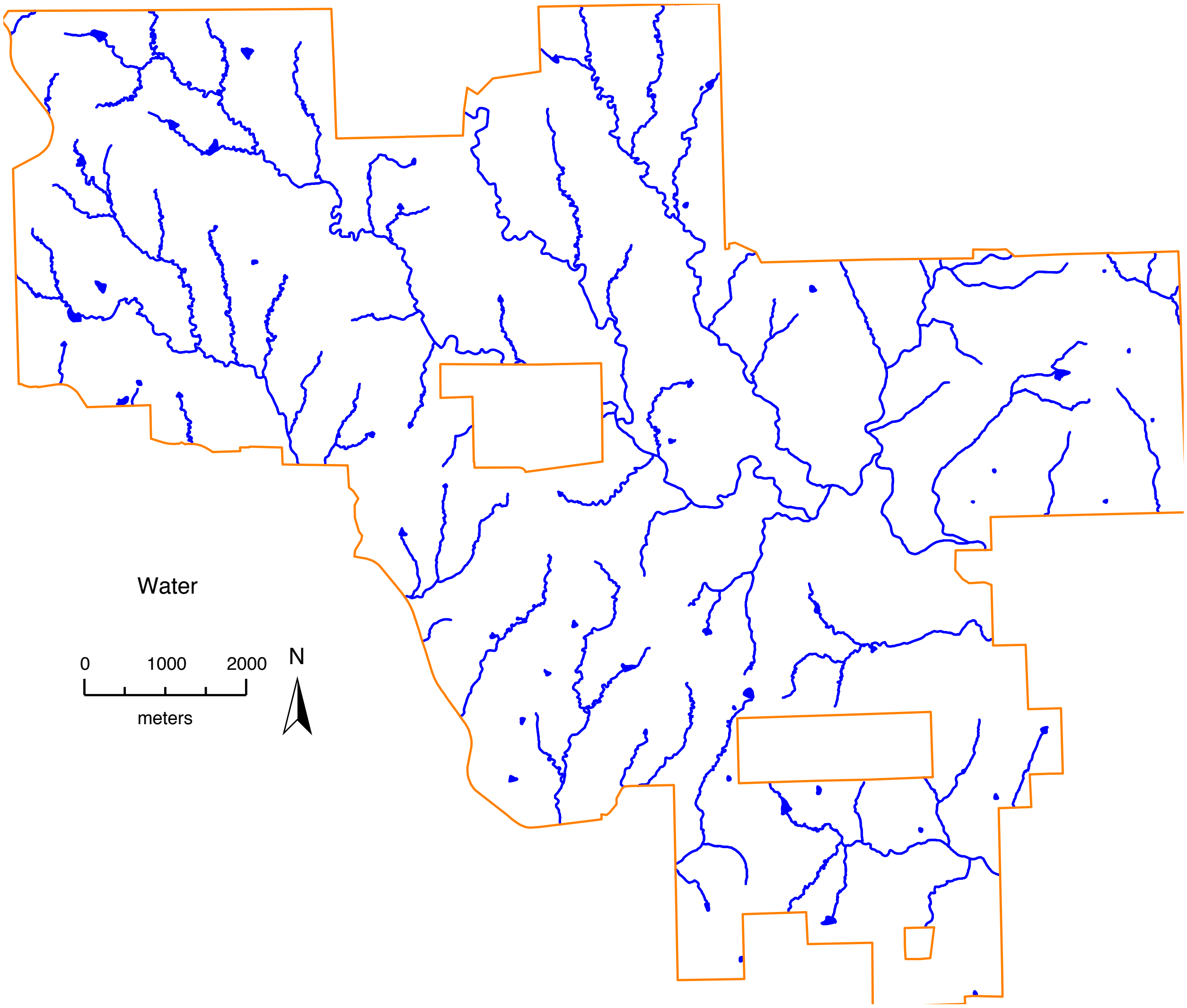


Figure S4: Water distribution (blue) within the the bison unit (9532 ha) at the Tallgrass Prairie Preserve, OK, USA. Solid orange lines represent perimeter fences.

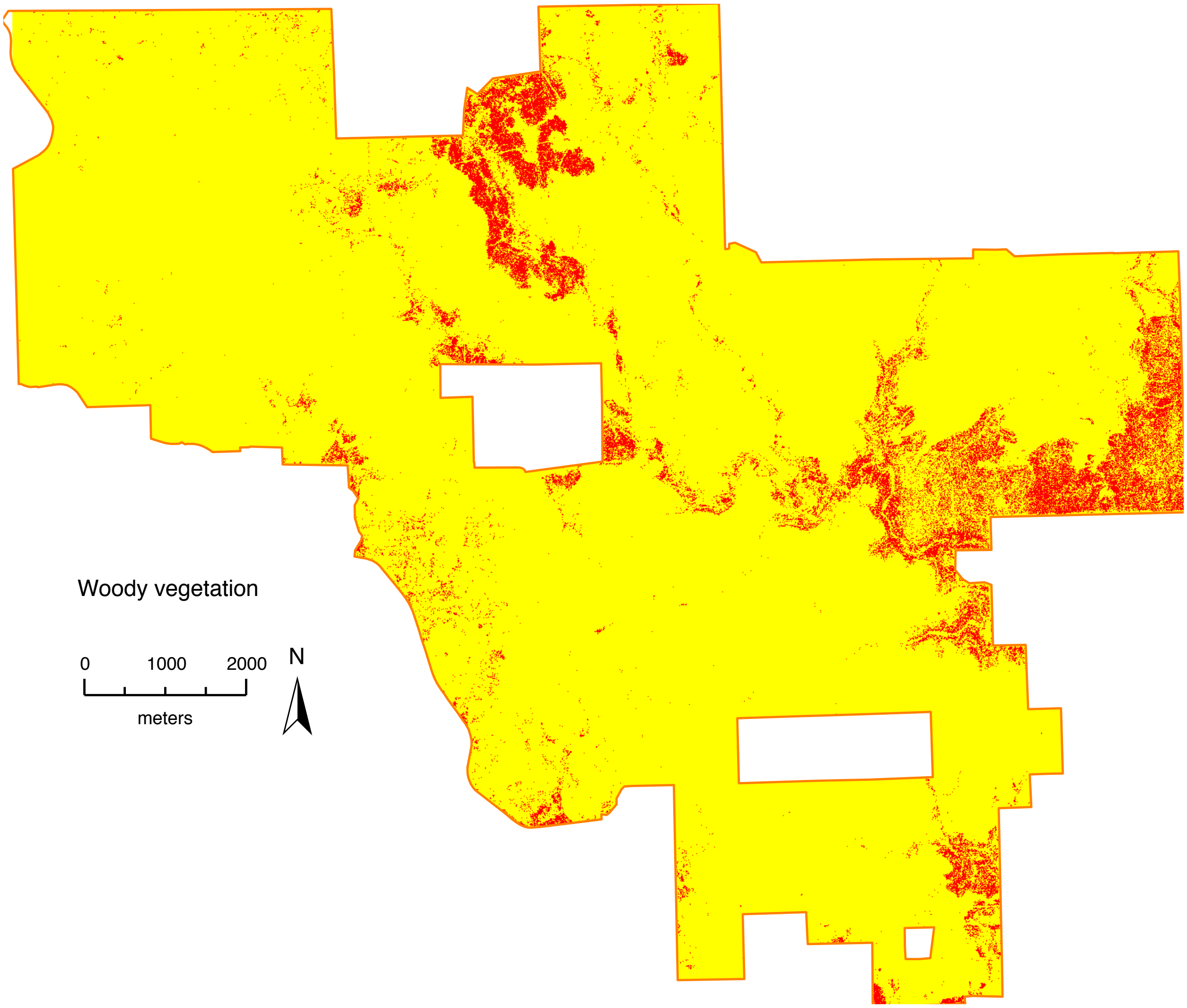


Figure S5: Woody vegetation (red) within the the bison unit (9532 ha) at the Tallgrass Prairie Preserve, OK, USA. Solid orange lines represent perimeter fences.
