# Supplementary material for: Ungulate preference for burned patches reveals strength of fire–grazing interaction
Source: Ecol Evol. 2011 Oct;1(2):132–44. doi: 10.1002/ece3.12 (PMC3287302; doi:10.1002/ece3.12)
Supplement: Supplementary file 2 [file ece30001-0132-SD2.pdf]

Table S1

The difference in Akaike information criterion (ΔAIC) and the number of parameters (K) for varying models of resource selection for bison at the Tallgrass Prairie Preserve, OK, USA; model parameters include time since fire (*tsf*), distance to water (*water*), slope (*slope*), distance to fire patch edge (*edge*), woody vegetation (*wood*), elevation (*elevation)*, fire patch area (*area*), northing (*north*) and easting (*east*), both derivatives of aspect. We included individual animals as a random intercept in the mixed-effect logistic regression. We included main effects in all models with interaction terms.

	K	ΔAIC	LL
tsf×water + tsf×slope + tsf×edge + tsf×wood + tsf×elevation + tsf×area + tsf×north + tsf×east	19	8.6	-627312.1
tsf×water + tsf×slope + tsf×edge + tsf×wood + tsf×elevation + tsf×area + north + east	17	16.0	-627317.8
tsf×water + tsf×slope + tsf×edge + tsf×wood + tsf×elevation + tsf×area	15	0.0	-627311.8
tsf×water + tsf×slope + tsf×edge + tsf×wood + tsf×elevation + area	14	9974.4	-632300.0
tsf×water + tsf×slope + tsf×edge + tsf×wood + elevation + tsf×area	14	2349.8	-628487.7
tsf×water + tsf×slope + tsf×edge + wood + tsf×elevation + tsf×area	14	15.8	-627320.7
tsf×water + tsf×slope + edge + tsf×wood + tsf×elevation + tsf×area	14	1431.4	-628028.5
tsf×water + slope + tsf×edge + tsf×wood + tsf×elevation + tsf×area	14	1533.4	-628079.5
water + tsf×slope + tsf×edge + tsf×wood + tsf×elevation + tsf×area	14	1477.0	-628051.3
tsf + water + slope + edge + wood + elevation + area	9	18583.8	-636609.7
water + slope + edge + wood + elevation + area	8	68313.4	-661475.5

Table S2

The difference in Akaike information criterion (ΔAIC) and the number of parameters (K) for varying models of resource selection for cattle at the Tallgrass Prairie Preserve, OK, USA; model parameters include time since fire (*tsf*), distance to water (*water*), slope (*slope*), distance to fire patch edge (*edge*), woody vegetation (*wood*), elevation (*elevation)*, northing (*north*) and easting (*east*), both derivatives of aspect. We included individual animals as a random intercept in the mixed-effect logistic regression. We included main effects in all models with interaction terms.

	K	ΔAIC	LL
tsf×water + tsf×slope + tsf×edge + tsf×wood + tsf×elevation + tsf×north + tsf×east	18	1.2	-466100.2
tsf×water + tsf×slope + tsf×edge + tsf×wood + tsf×elevation + north + east	16	0.0	-466101.6
tsf×water + tsf×slope + tsf×edge + tsf×wood + tsf×elevation	14	45.4	-466126.3
tsf×water + tsf×slope + tsf×edge + tsf×wood + tsf×elevation	14	45.4	-466126.3
tsf×water + tsf×slope + tsf×edge + tsf×wood + elevation	13	157.0	-466183.1
tsf×water + tsf×slope + tsf×edge + wood + tsf×elevation	13	352.2	-466280.7
tsf×water + tsf×slope + edge + tsf×wood + tsf×elevation	13	183.8	-466196.5
tsf×water + slope + tsf×edge + tsf×wood + tsf×elevation	13	1647.8	-466928.5
water + tsf×slope + tsf×edge + tsf×wood + tsf×elevation	13	1761.6	-466985.4
tsf + water + slope + edge + wood + elevation	9	4406.4	-468311.8
water + slope + edge + wood + elevation	8	38629.0	-48542.1

Table S3

The difference in Akaike information criterion (ΔAIC) and the number of parameters (K) for varying models of resource selection for cattle at the Oklahoma State University Research Range, OK, USA; model parameters include time since fire (*tsf*), distance to water (*water*), slope (*slope*), woody vegetation (*wood*), northing (*north*) and easting (*east*), both derivatives of aspect. We included individual animals as a random intercept in the mixed-effect logistic regression. We included main effects in all models with interaction terms.

	K	ΔAIC	LL
tsf×water + tsf×slope + tsf×wood + tsf×north + tsf×east	12	4.6	-415563.5
tsf×water + tsf×slope + tsf×wood + north + east	10	0.0	-415563.2
tsf×water + tsf×slope + tsf×wood	8	71.8	-415581.6
tsf×water + tsf×slope + wood	7	768.0	-415930.7
tsf×water + slope + tsf×wood	7	148.0	-415620.8
water + tsf×slope + tsf×wood	7	240.1	-415666.8
tsf + water + slope + wood	5	953.5	-416025.5
water + slope + wood	4	66650.8	0.0

Table S4
Varying models of resource selection for cattle at the Tallgrass Prairie Preserve, OK, USA. Patches in cattle units vary in size relative to total area. The difference in Akaike information criterion (ΔAIC), log likelihood (LL), and the number of parameters (K) are shown. Model parameters include time since fire (*tsf*), distance to water (*water*), slope (*slope*), distance to fire patch edge (*edge*), woody vegetation (*wood*), elevation (*elevation)*, northing (*north*) and easting (*east*), both derivatives of aspect. We included individual animals nested within fire patches as a random intercept in the mixed-effect logistic regression. We included main effects in all models with interaction terms.

	K	ΔAIC	LL
**Patch size - 50%**			
tsf×water + tsf×slope + tsf×edge + tsf×wood + tsf×elevation + tsf×north + tsf×east	17	5.8	-33925.83
tsf×water + tsf×slope + tsf×edge + tsf×wood + tsf×elevation + north + east	15	3.1	-33926.46
tsf×water + tsf×slope + tsf×edge + tsf×wood + tsf×elevation	13	3.0	-33928.43
tsf×water + tsf×slope + tsf×edge + tsf×wood + elevation	12	51.1	-33953.48
tsf×water + tsf×slope + tsf×edge + wood + tsf×elevation	12	2.2	-33929.00
tsf×water + tsf×slope + edge + tsf×wood + tsf×elevation	12	514.9	-34185.37
tsf×water + slope + tsf×edge + tsf×wood + tsf×elevation	12	30.4	-33943.11
water + tsf×slope + tsf×edge + tsf×wood + tsf×elevation	12	0.0	-33927.92
tsf + water + slope + edge + wood + elevation	8	744.6	-34304.20
**Patch size - 33%**			
tsf×water + tsf×slope + tsf×edge + tsf×wood + tsf×elevation + tsf×north + tsf×east	17	8.4	-86403.15
tsf×water + tsf×slope + tsf×edge + tsf×wood + tsf×elevation + north + east	15	0.0	-86400.95
tsf×water + tsf×slope + tsf×edge + tsf×wood + tsf×elevation	13	21.7	-86413.80
tsf×water + tsf×slope + tsf×edge + tsf×wood + elevation	12	36.2	-86422.05
tsf×water + tsf×slope + tsf×edge + wood + tsf×elevation	12	72.1	-86440.00
tsf×water + tsf×slope + edge + tsf×wood + tsf×elevation	12	1507.0	-87157.45
tsf×water + slope + tsf×edge + tsf×wood + tsf×elevation	12	11.1	-86409.50
water + tsf×slope + tsf×edge + tsf×wood + tsf×elevation	12	42.4	-86425.15
tsf + water + slope + edge + wood + elevation	8	2453.9	-87484.90
**Patch size - 25%**			
tsf×water + tsf×slope + tsf×edge + tsf×wood + tsf×elevation + tsf×north + tsf×east	17	9.6	-101297.40
tsf×water + tsf×slope + tsf×edge + tsf×wood + tsf×elevation + north + east	15	3.5	-101296.35
tsf×water + tsf×slope + tsf×edge + tsf×wood + tsf×elevation	13	0.0	-101296.60
tsf×water + tsf×slope + tsf×edge + tsf×wood + elevation	12	4.5	-101299.85
tsf×water + tsf×slope + tsf×edge + wood + tsf×elevation	12	72.7	-101333.95
tsf×water + tsf×slope + edge + tsf×wood + tsf×elevation	12	1749.5	-101672.35
tsf×water + slope + tsf×edge + tsf×wood + tsf×elevation	12	8.7	-101301.95
water + tsf×slope + tsf×edge + tsf×wood + tsf×elevation	12	113.0	-101354.10
tsf + water + slope + edge + wood + elevation	8	955.2	-101779.20
**Patch size - 17%**			
tsf×water + tsf×slope + tsf×edge + tsf×wood + tsf×elevation + tsf×north + tsf×east	17	0.0	-92030.60
tsf×water + tsf×slope + tsf×edge + tsf×wood + tsf×elevation + north + east	15	28.6	-92043.90

tsf×water + tsf×slope + tsf×edge + tsf×wood + tsf×elevation	13	77.4	-92.073.3
tsf×water + tsf×slope + tsf×edge + tsf×wood + elevation	12	308.7	-92189.95
tsf×water + tsf×slope + tsf×edge + wood + tsf×elevation	12	80.3	-92075.72
tsf×water + tsf×slope + edge + tsf×wood + tsf×elevation	12	5404.6	-94737.90
tsf×water + slope + tsf×edge + tsf×wood + tsf×elevation	12	87.9	-92079.55
water + tsf×slope + tsf×edge + tsf×wood + tsf×elevation	12	134.3	-92102.75
tsf + water + slope + edge + wood + elevation	8	6050.6	-95064.90
**Patch size - 12%**			
tsf×water + tsf×slope + tsf×edge + tsf×wood + tsf×elevation + tsf×north + tsf×east	17	1.2	-47284.00
tsf×water + tsf×slope + tsf×edge + tsf×wood + tsf×elevation + north + east	15	0.0	-47285.39
tsf×water + tsf×slope + tsf×edge + tsf×wood + tsf×elevation	13	0.3	-47287.52
tsf×water + tsf×slope + tsf×edge + tsf×wood + elevation	12	168.2	-47372.47
tsf×water + tsf×slope + tsf×edge + wood + tsf×elevation	12	20.2	-47298.49
tsf×water + tsf×slope + edge + tsf×wood + tsf×elevation	12	198.6	-47387.68
tsf×water + slope + tsf×edge + tsf×wood + tsf×elevation	12	49.1	-47312.92
water + tsf×slope + tsf×edge + tsf×wood + tsf×elevation	12	132.5	-47351.62
tsf + water + slope + edge + wood + elevation	8	477.8	-47531.27

Table S5

List of studies that examine the fire-grazing interaction, separated by continent. List is not all inclusive.

Africa	
	Moe, Wegge, & Kapela 1990; Wilsey 1996; Salvatori et al. 2001; Gureja & Owen-Smith 2002; Tomor & Owen-Smith 2002; Archibald & Bond 2004; Archibald et al. 2005; Klop, van Goethem, & de Iongh 2007; Savadogo, Sawadogo, & Tiveau 2007; Archibald 2008; Hassan et al. 2008; Klop & van Goethem 2008; Waldram, Bond, & Stock 2008; Parrini & Owen-Smith 2010
Asia	
	Moe & Wegge 1994; Moe & Wegge 1997; Sankaran 2005
Australia	
	Kirkpatrick, Marsden-Smedley, & Leonard In press; Kutt & Woinarski 2007; Murphy & Bowman 2007; Leonard, Kirkpatrick, & Marsden-Smedley 2010
Europe	
	Kramer, Groen, & van Wieren 2003; Vandvik et al. 2005; Onodi et al. 2008; Davies et al. 2010
North America	
	Duvall & Whitaker 1964; Hobbs & Spowart 1984; Vinton et al. 1993; Turner et al. 1994; Pearson et al. 1995; Wallace et al. 1995; Coppedge & Shaw 1998; Biondini, Steuter, & Hamilton 1999; Smith, Hardin, & Flinders 1999; Fuhlendorf & Engle 2004; Schuler et al. 2006; Van Dyke & Darragh 2007; Bleich et al. 2008; Meek et al. 2008

References for Table S5

Archibald, S. (2008) African grazing lawns - how fire, rainfall, and grazer numbers interact to affect grass community states. *Journal of Wildlife Management*, 72, 492-501.

Archibald, S. & Bond, W.J. (2004) Grazer movements: spatial and temporal responses to burning in a tall-grass African savanna. *International Journal of Wildland Fire*, 13, 377-385.

Archibald, S., Bond, W.J., Stock, W.D. & Fairbanks, D.H.K. (2005) Shaping the landscape: fire-grazer interactions in an African savanna. *Ecological Applications*, 15, 96-109.

Biondini, M.E., Steuter, A.A. & Hamilton, R.G. (1999) Bison use of fire-managed remnant prairies. *Journal of Range Management*, 52, 454-461.

Bleich, V.C., Johnson, H.E., Holl, S.A., Konde, L., Torres, S.G. & Krausman, P.R. (2008) Fire history in a Chaparral ecosystem: implications for conservation of a native ungulate. *Rangeland Ecology and Management*, 61, 571-579.

Coppedge, B.R. & Shaw, J.H. (1998) Bison grazing patterns on seasonally burned tallgrass prairie. *Journal of Range Management*, 51, 258-264.

Davies, M., Smith, A., MacDonald, A.J., Bakker, J.D. & Legg, C.J. (2010) Fire intensity, fire severity and ecosystem response in heathlands: factors affecting the regeneration of Calluna vulgaris. *Journal of Applied Ecology*, 47, 356-365.

Duvall, V.L. & Whitaker, L.B. (1964) Rotation burning: a forage management system for longleaf pine-bluestem ranges. *Journal of Range Management*, 17, 322-326.

Fuhlendorf, S.D. & Engle, D.M. (2004) Application of the fire-grazing interaction to restore a shifting mosaic on tallgrass prairie. *Journal of Applied Ecology*, 41, 604-614.

Gureja, N. & Owen-Smith, N. (2002) Comparative use of burnt grassland by rare antelope species in a lowveld game ranch, South Africa. *South African Journal of Wildlife Research*, 32, 31-38.

Hassan, S.N., Rusch, G.M., Hytteborn, H., Skarpe, C. & Kikula, I. (2008) Effects of fire on sward structure and grazing in western Serengeti, Tanzania. *African Journal of Ecology*, 46, 174-185.

Hobbs, N.T. & Spowart, R.A. (1984) Effects of prescribed fire on nutrition of mountain sheep and mule deer during winter and spring. *The Journal of Wildlife Management*, 48, 551-560.

Kirkpatrick, J.B., Marsden-Smedley, J.B. & Leonard, S.W.J. (In press) Influence of grazing and vegetation type on post-fire flammability. *Journal of Applied Ecology*.

Klop, E. & van Goethem, J. (2008) Savanna fires govern community structure of ungulates in Benoue National Park, Cameroon. *Journal of Tropical Ecology*, 24, 39-47.

Klop, E., van Goethem, J. & de Iongh, H.H. (2007) Resource selection by grazing herbivores on post-fire regrowth in a West African woodland savanna. *Wildlife Research*, 34, 77-83.

Kramer, K., Groen, T.A. & van Wieren, S.E. (2003) The interacting effects of ungulates and fire on forest dynamics: an analysis using the model FORSPACE. *Forest Ecology and Management*, 181, 205-222.

Kutt, A.S. & Woinarski, J.C.Z. (2007) The effects of grazing and fire on vegetation and the vertebrate assemblage in a tropical savanna woodland in north-eastern Australia. *Journal of Tropical Ecology*, 23, 95-106.

Leonard, S., Kirkpatrick, J. & Marsden-Smedley, J. (2010) Variation in the effects of vertebrate grazing on fire potential between grassland structural types. *Journal of Applied Ecology*, 47, 876-883.

Meek, M.G., Cooper, S.M., Owens, M.K., Cooper, R.M. & Wappel, A.L. (2008) White-tailed deer distribution in response to patch burning on rangeland. *Journal of Arid Environments*, 72, 2026-2033.

Moe, S.R. & Wegge, P. (1997) The effects of cutting and burning on grass quality and axis deer (Axis axis) use of grassland in lowland Nepal. *Journal of Tropical Ecology*, 13, 279-292.

Moe, S.R., Wegge, P. & Kapela, E.B. (1990) The influence of man-made fires on large wild herbivores in Lake Burungi area in northern Tanzania. *African Journal of Ecology*, 28, 35-43.

Moe, S.R. & Wegge, P. (1994) Spacing behaviour and habitat use of axis deer (Axis axis) in lowland Nepal. *Canadian Journal of Zoology*, 72, 1735-1744.

Murphy, B.P. & Bowman, D.M.J.S. (2007) The interdependence of fire, grass, kangaroos and Australian Aborigines: a case study from central Arnhem Land, northern Australia. *Journal of Biogeography*, 34, 237-250.

Onodi, G., Kertesz, M., Botta-Dukat, Z. & Altbacker, V. (2008) Grazing effects on vegetation composition and on the spread of fire on open sand grasslands. *Arid Land Research and Management*, 22, 273-285.

Parrini, F. & Owen-Smith, N. (2010) The importance of post-fire regrowth for sable antelope in a Southern African savanna. *African Journal of Ecology*, 48, 526-534.

Pearson, S.M., Turner, M.G., Wallace, L.L. & Romme, W.H. (1995) Winter habitat use by large ungulates following fire in northern Yellowstone National Park. *Ecological Applications*, 5, 744-755.

Salvatori, R., Egunyu, F., Skidmore, A.K., de Leeuw, J. & van Gils, H.A.M. (2001) The effects of fire and grazing pressure on vegetation cover and small mammal populations in the Maasai Mara National Reserve. *African Journal of Ecology*, 39, 200-204.

Sankaran, M. (2005) Fire, Grazing and the Dynamics of Tall-Grass Savannas in the Kalakad-Mundanthurai Tiger Reserve, South India. *Conservation and Society*, 3, 4-25.

Savadogo, P., Sawadogo, L. & Tiveau, D. (2007) Effects of grazing intensity and prescribed fire on soil physical and hydrological properties and pasture yield in the savanna woodlands of Burkina Faso. *Agriculture, Ecosystems & Environment*, 118, 80-92.

Schuler, K.L., Leslie, D.M., Shaw, J.H. & Maichak, E.J. (2006) Temporal-spatial distribution of American bison (Bison bison) in a tallgrass prairie fire mosaic. *Journal of Mammalogy*, 87, 539-544.

Smith, T.S., Hardin, P.J. & Flinders, J.T. (1999) Response of bighorn sheep to clear-cut logging and prescribed burning. *Wildlife Society Bulletin*, 27, 840-845.

Tomor, B.M. & Owen-Smith, N. (2002) Comparative use of grass regrowth following burns by four ungulate species in the Nylsvley Nature Reserve, South Africa. *African Journal of Ecology*, 40, 201-204.

Turner, M.G., Wu, Y.A., Wallace, L.L., Romme, W.H. & Brenkert, A. (1994) Simulating winter interactions among ungulates, vegetation, and fire in northern Yellowstone park. *Ecological Applications*, 4, 472-486.

Vandvik, V., Heegaard, E., Maren, I.E. & Aarrestad, P.A. (2005) Managing heterogeneity: the importance of grazing and environmental variation on post-fire succession in heathlands. *Journal of Applied Ecology*, 42, 139-149.

Van Dyke, F. & Darragh, J.A. (2007) Response of elk to changes in plant production and nutrition following prescribed burning. *Journal of Wildlife Management*, 71, 23-29.

Vinton, M.A., Hartnett, D.C., Finck, E.J. & Briggs, J.M. (1993) Interactive effects of fire, bison (Bison bison) grazing and plant community composition in tallgrass prairie. *American Midland Naturalist*, 129, 10-18.

Waldram, M.S., Bond, W.J. & Stock, W.D. (2008) Ecological engineering by a mega-grazer: White Rhino impacts on a South African savanna. *Ecosystems*, 11, 101-112.

Wallace, L.L., Turner, M.G., Romme, W.H., Oneill, R.V. & Wu, Y.G. (1995) Scale of heterogeneity of forage production and winter foraging by elk and bison. *Landscape Ecology*, 10, 75-83.

Wilsey, B.J. (1996) Variation in use of green flushes following burns among African ungulate species: the importance of body size. *African Journal of Ecology*, 34, 32-38.
